# Supplementary figures and images for: TaZFP 23, a new Cys2/His2-type zinc-finger protein, is a regulator of wheat (Triticum aestivum L.) growth and abiotic stresses
Source: PeerJ. 2025 Feb 17;13:e18956. doi: 10.7717/peerj.18956 (PMC11841591; doi:10.7717/peerj.18956)

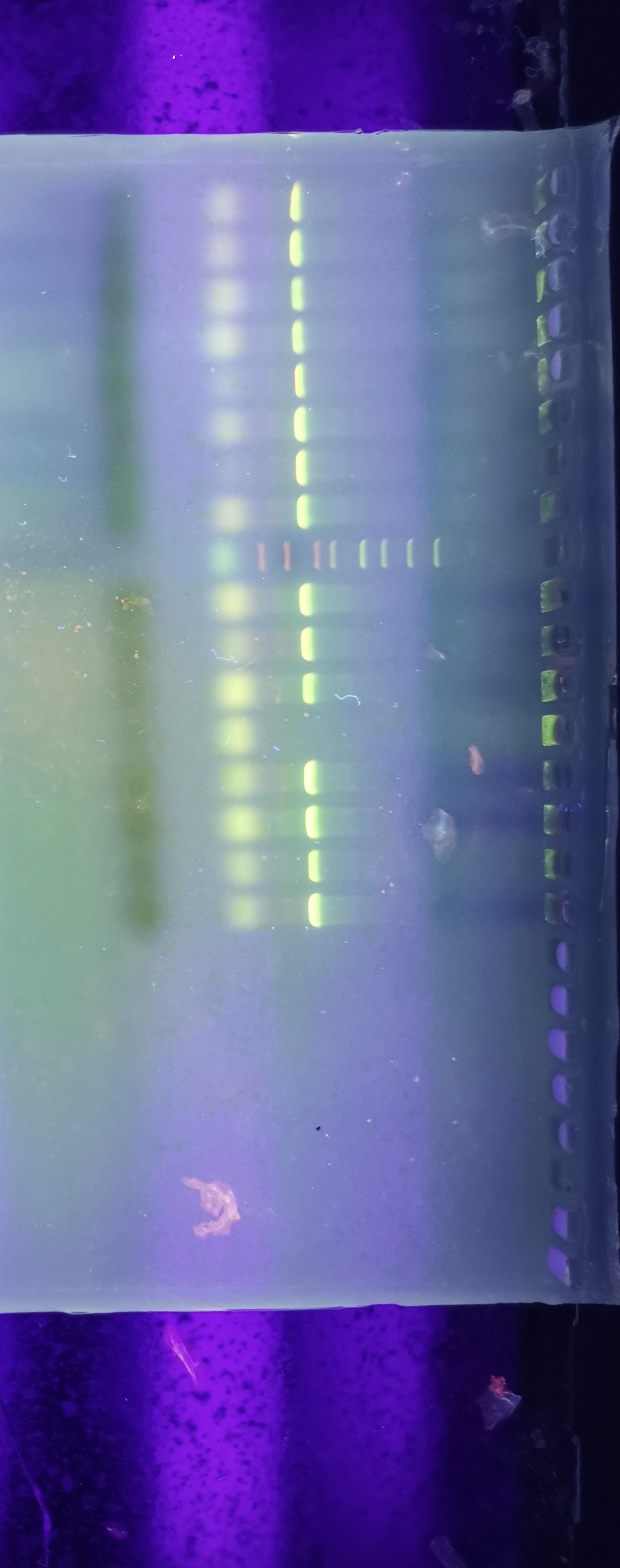

Supplement: Data S1 [file peerj-13-18956-s001.zip › Figure 1C Raw Data.jpg]

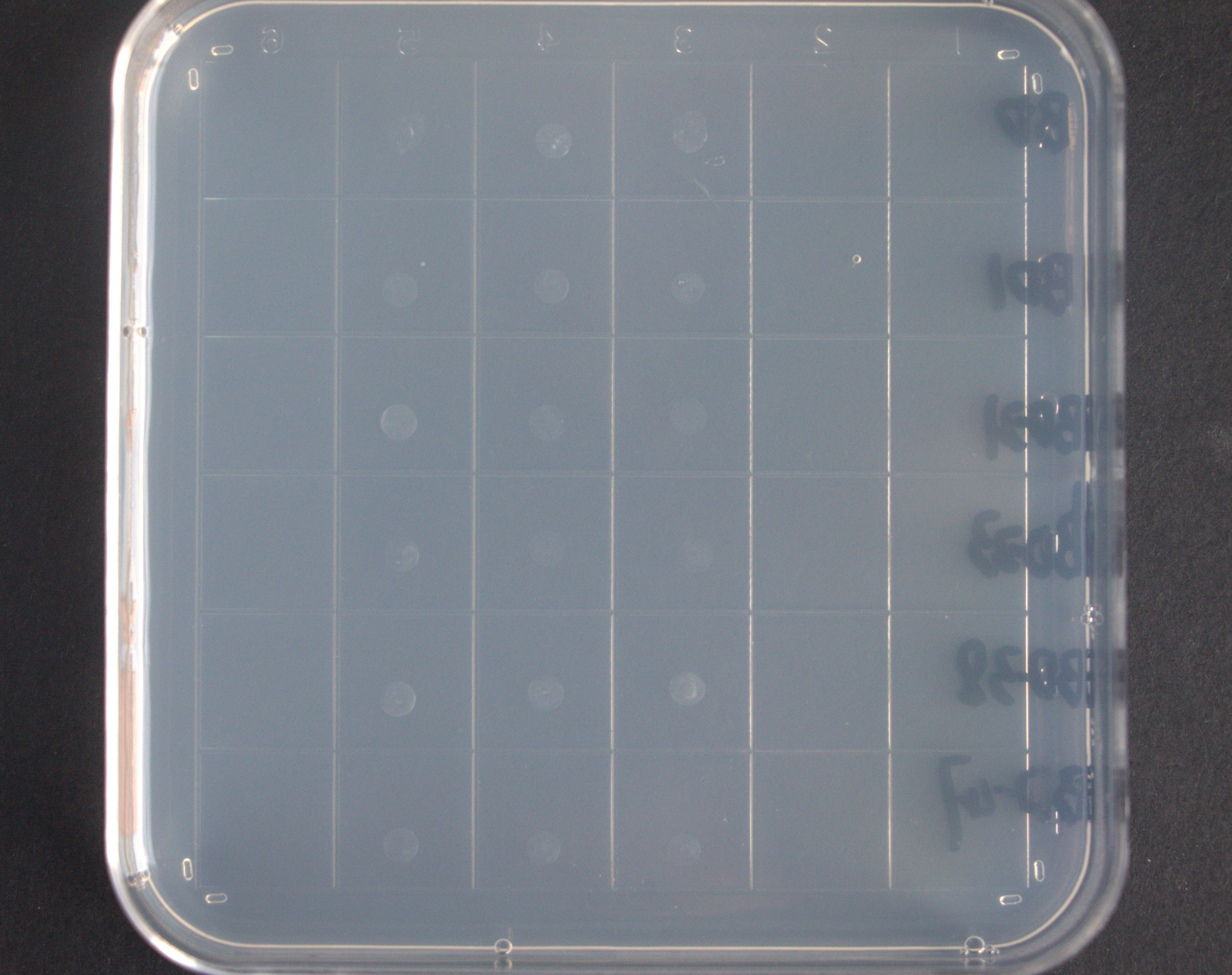

Supplement: Data S2 [file peerj-13-18956-s002.zip › Figure 4B Raw Data/Figure 4B-1.jpg]

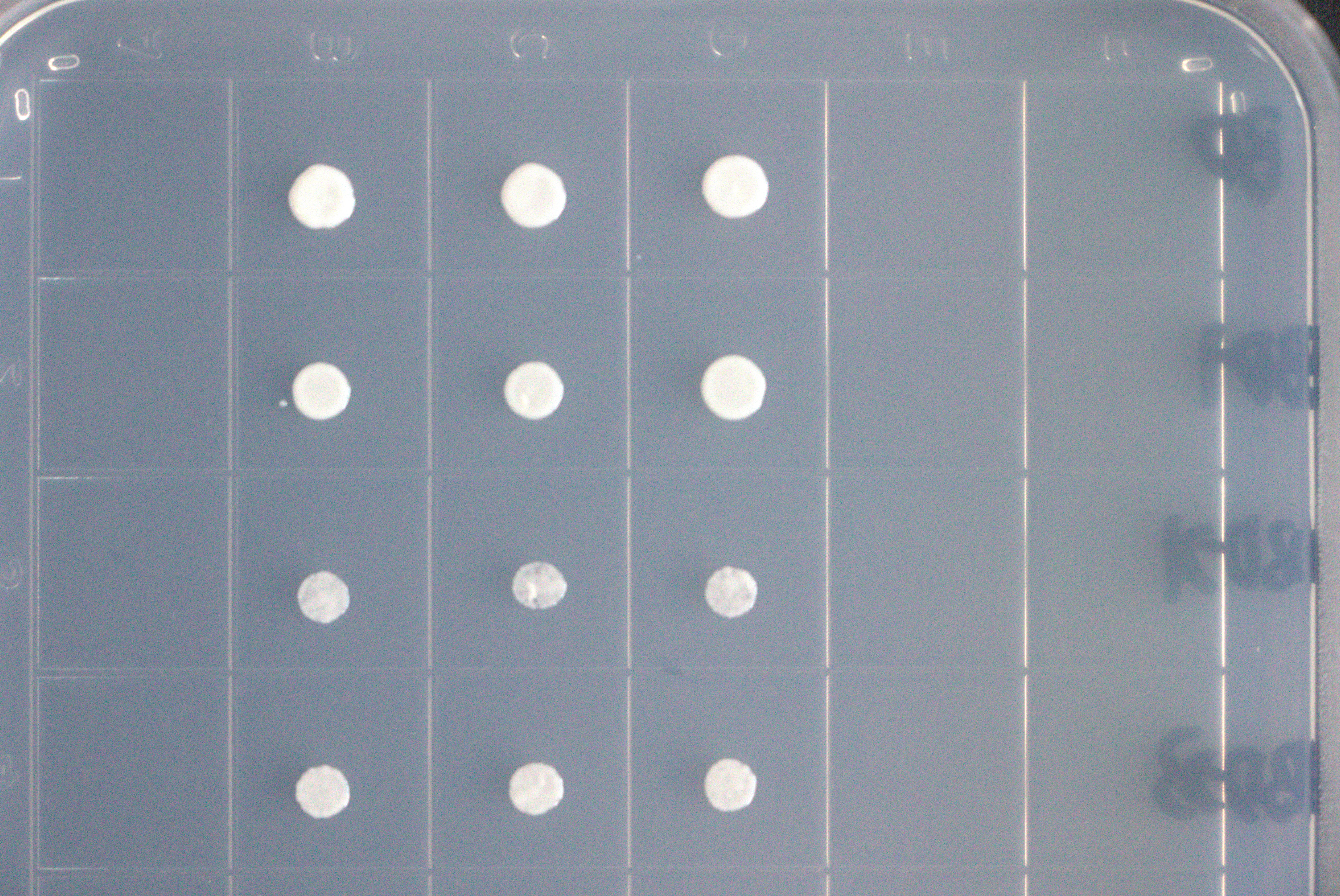

Supplement: Data S2 [file peerj-13-18956-s002.zip › Figure 4B Raw Data/Figure 4B-2.jpg]

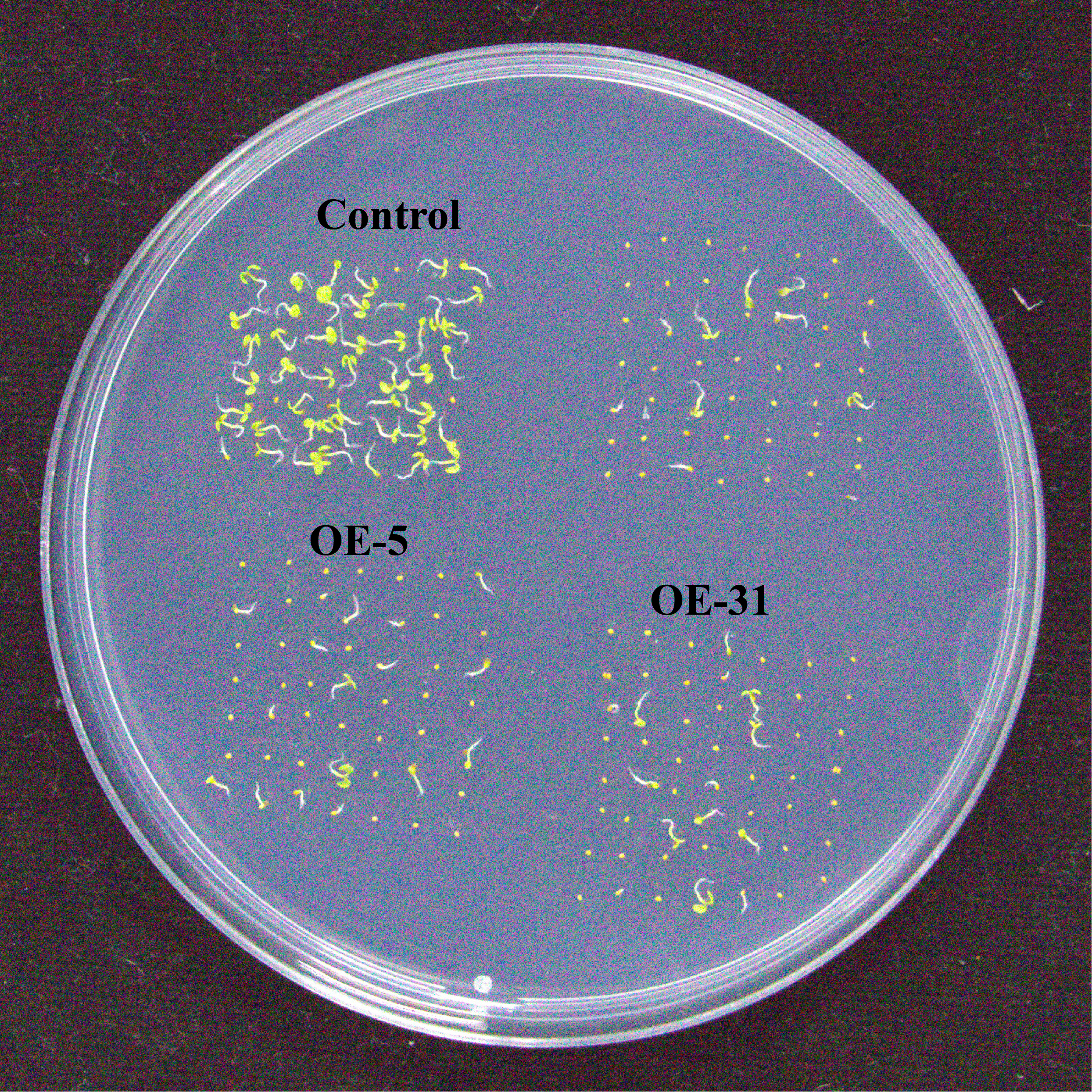

Supplement: Data S3 [file peerj-13-18956-s003.zip › Figure 5A+B-Nacl-Raw data/120mM-Nacl-2.jpg]

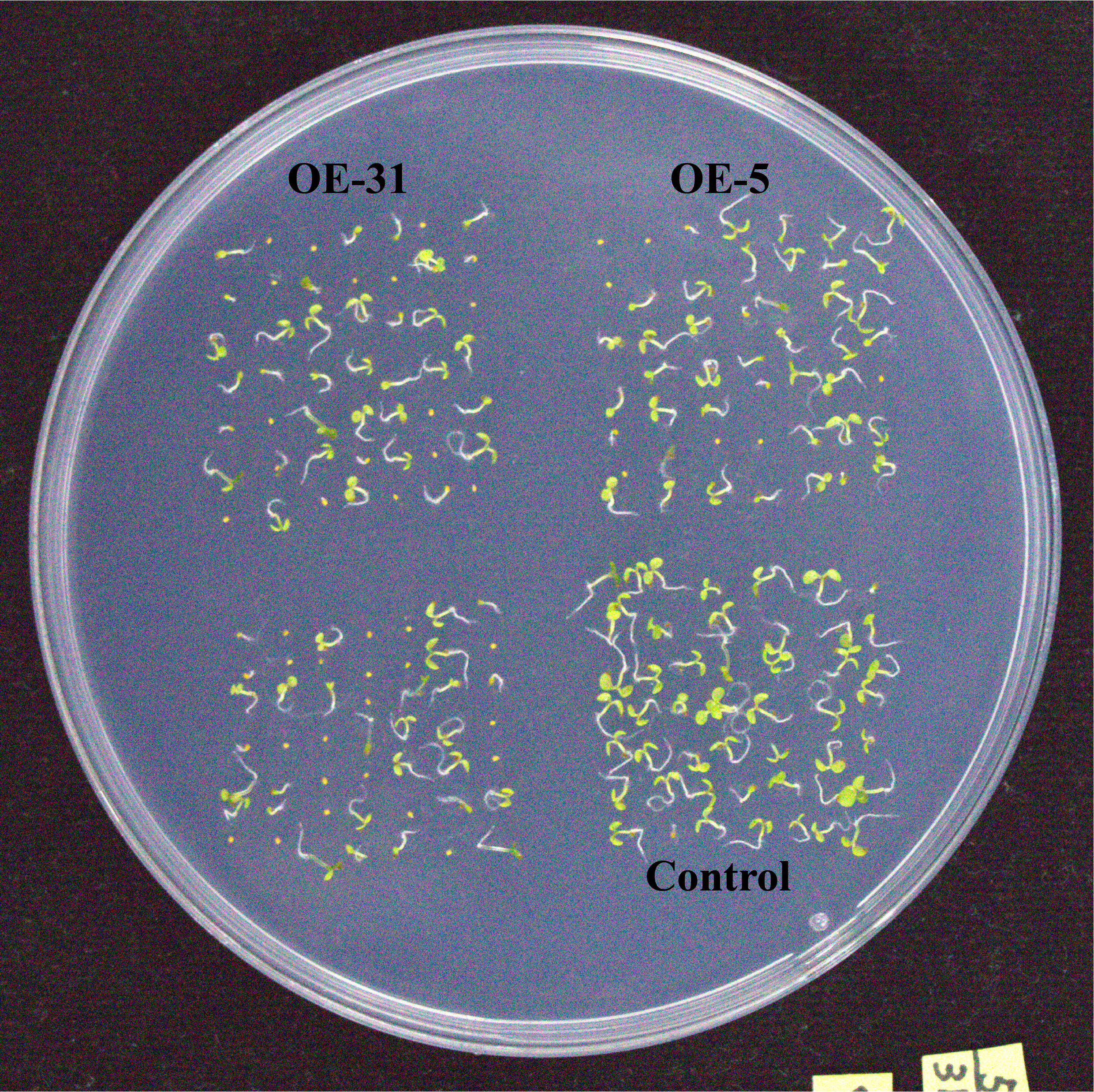

Supplement: Data S3 [file peerj-13-18956-s003.zip › Figure 5A+B-Nacl-Raw data/80mM-Nacl.jpg]

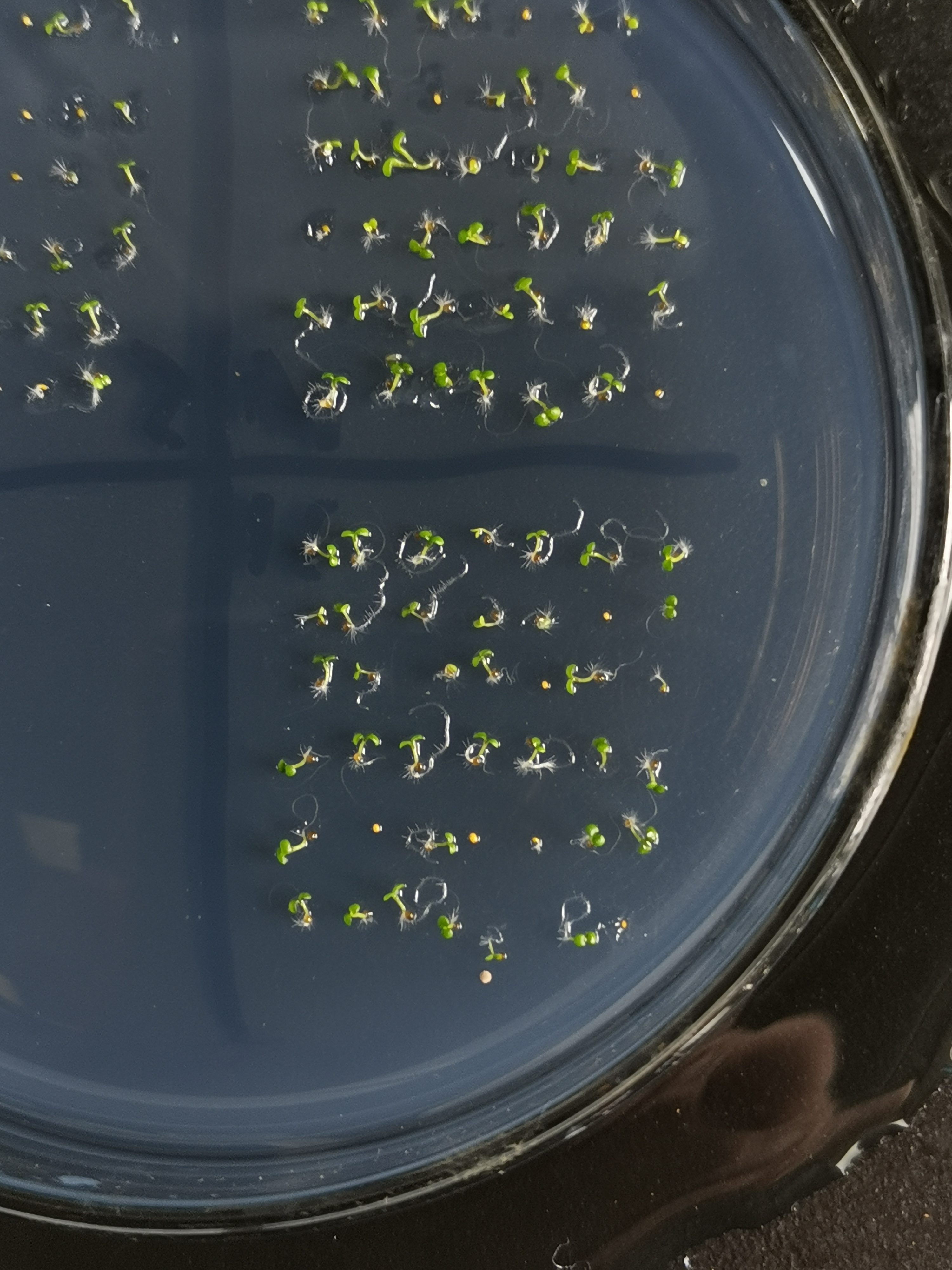

Supplement: Data S4 [file peerj-13-18956-s004.zip › Figure 5C+D-Mannitol-Raw data/200mM-Mannitol-31.jpg]

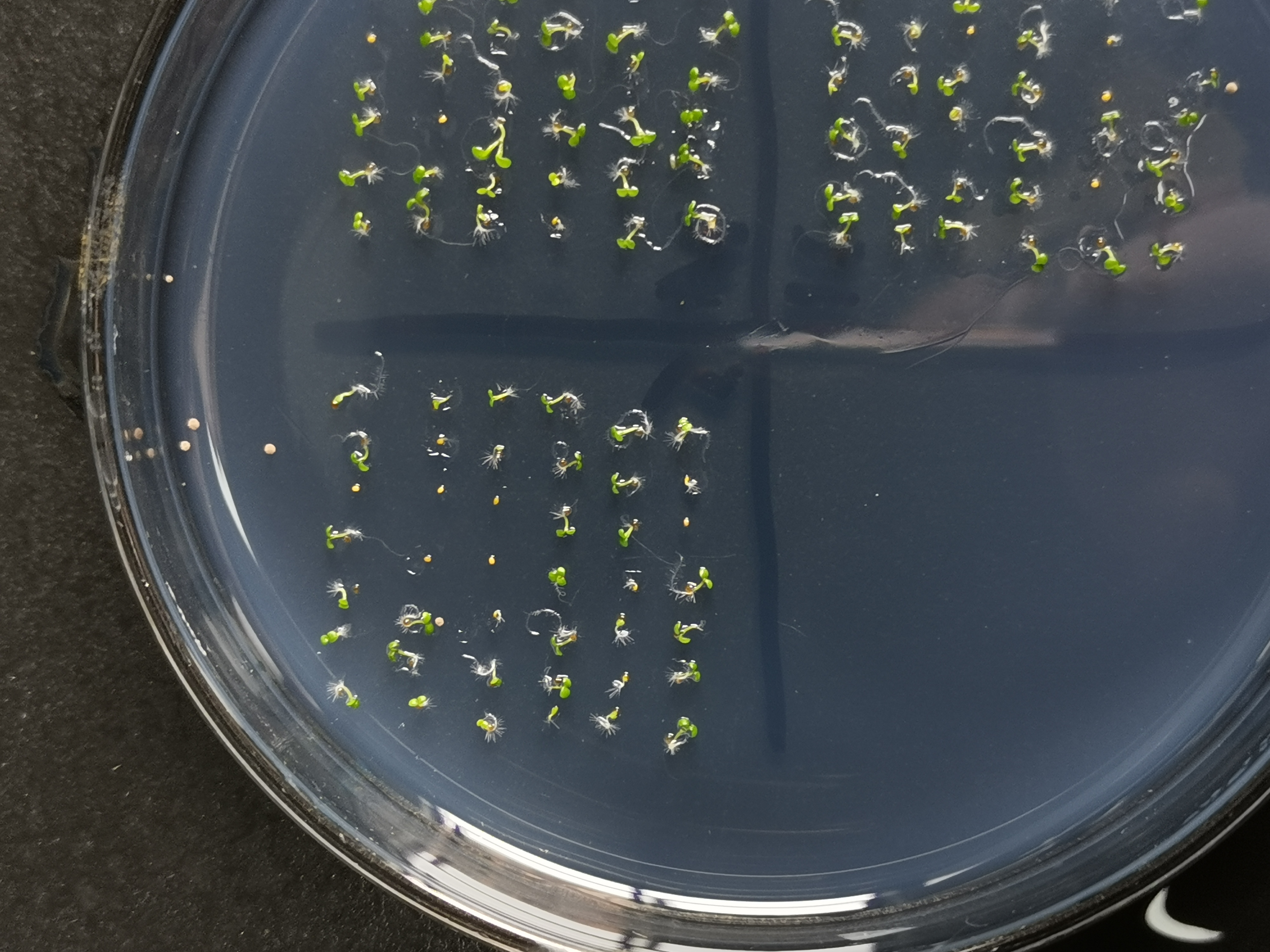

Supplement: Data S4 [file peerj-13-18956-s004.zip › Figure 5C+D-Mannitol-Raw data/200mM-Mannitol-5.jpg]

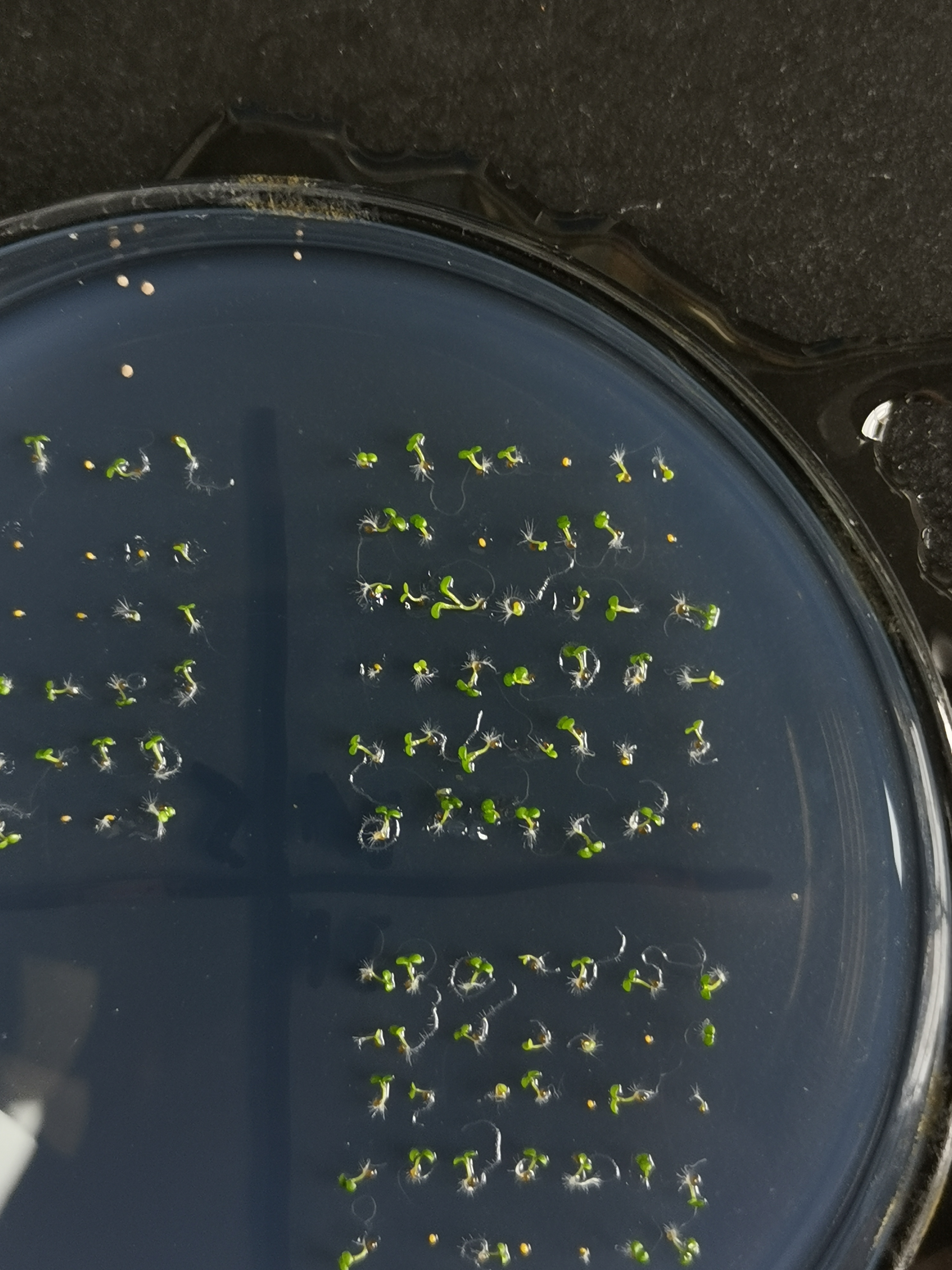

Supplement: Data S4 [file peerj-13-18956-s004.zip › Figure 5C+D-Mannitol-Raw data/200mM-Mannitol-WT.jpg]

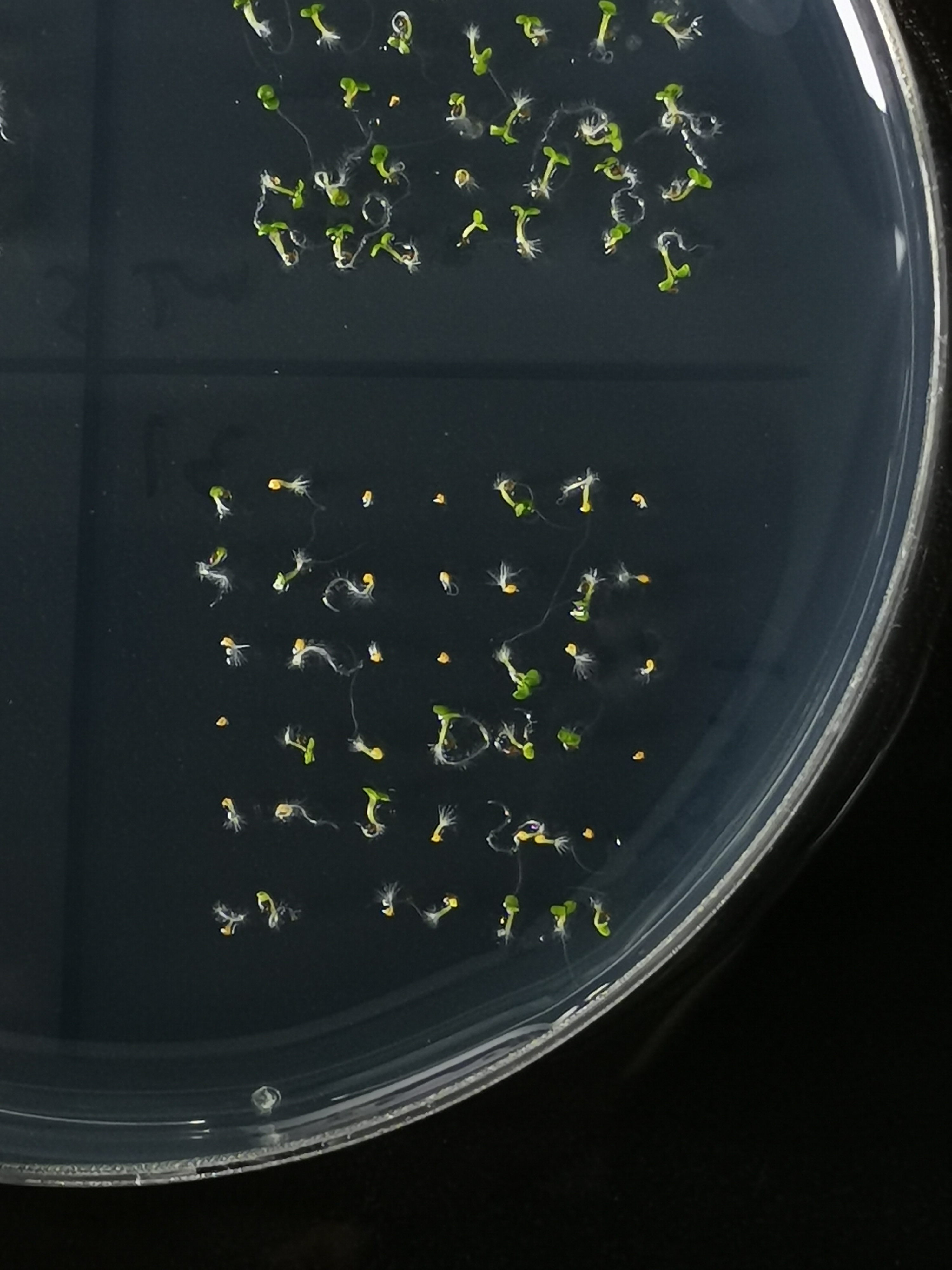

Supplement: Data S4 [file peerj-13-18956-s004.zip › Figure 5C+D-Mannitol-Raw data/250mM-Mannitol-31.jpg]

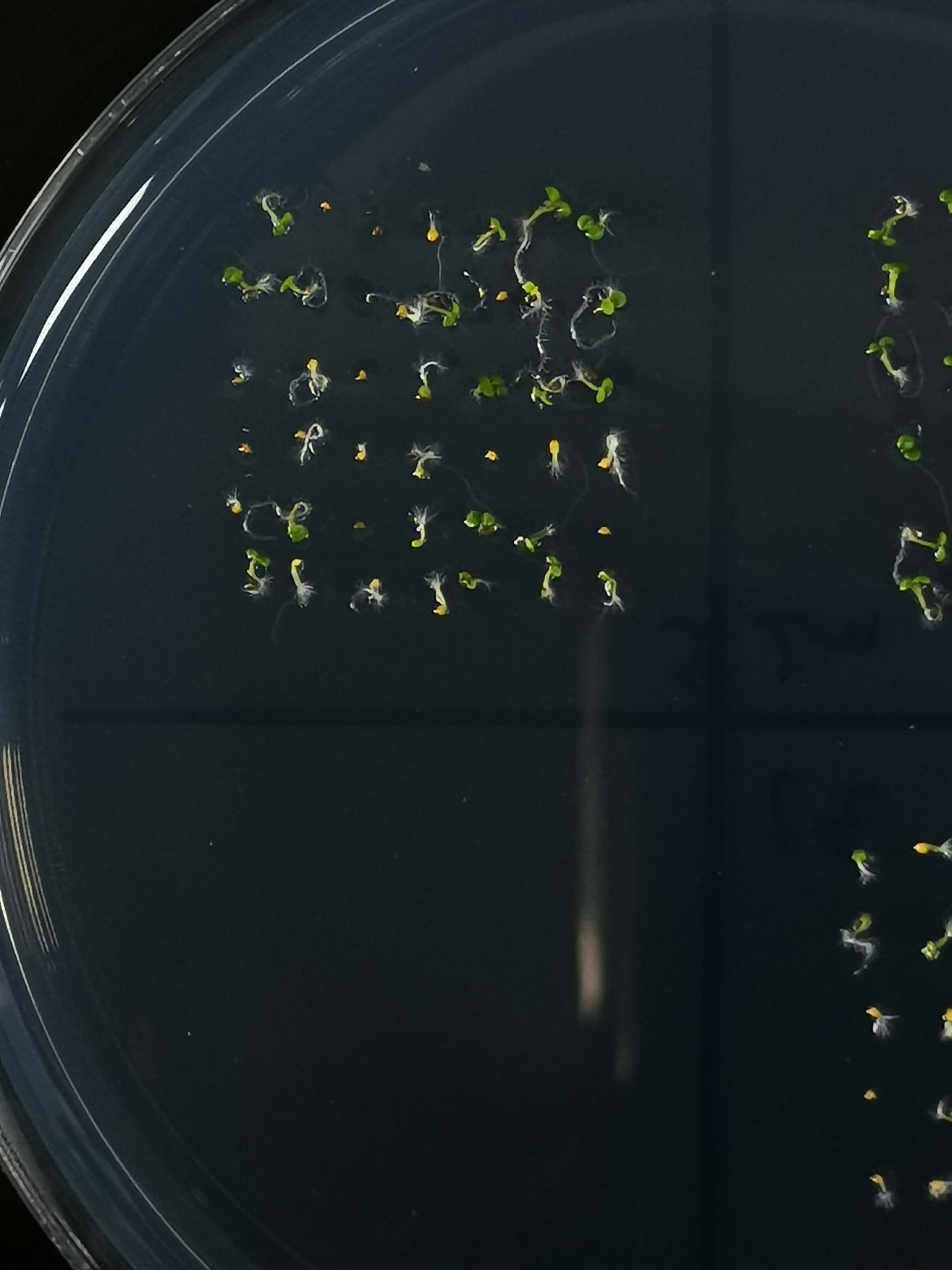

Supplement: Data S4 [file peerj-13-18956-s004.zip › Figure 5C+D-Mannitol-Raw data/250mM-Mannitol-5.jpg]

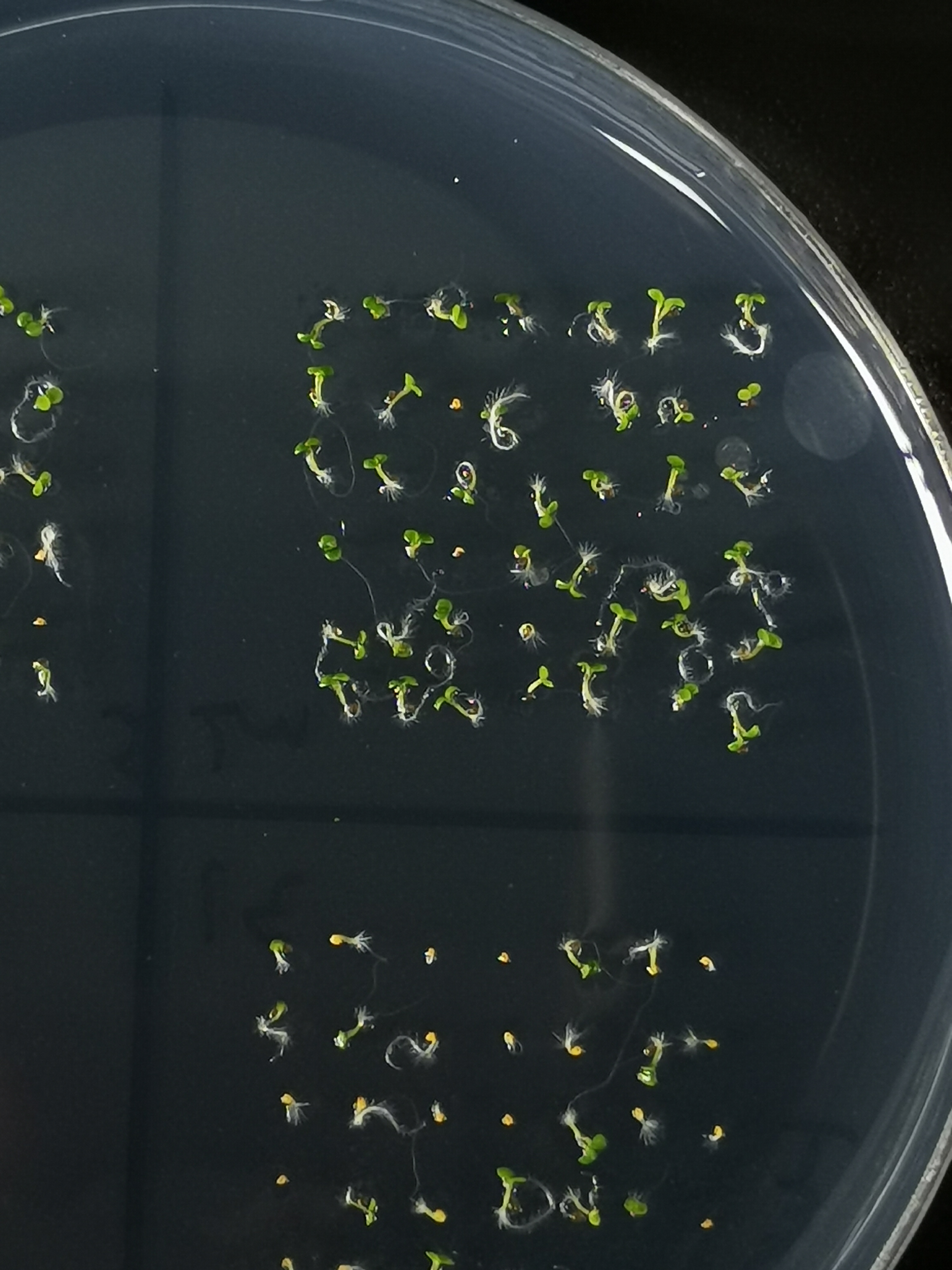

Supplement: Data S4 [file peerj-13-18956-s004.zip › Figure 5C+D-Mannitol-Raw data/250mM-MannitolWT.jpg]

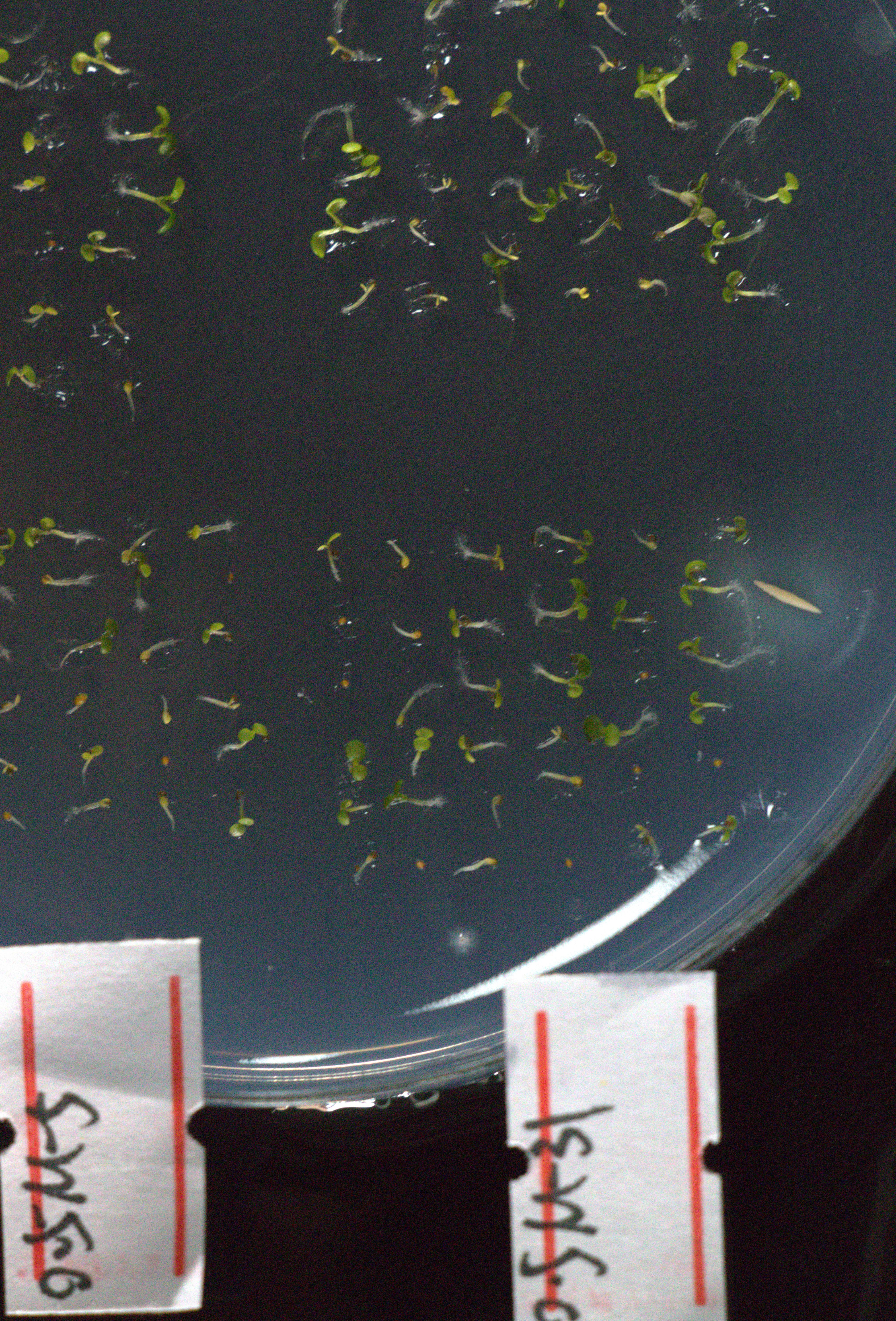

Supplement: Data S5 [file peerj-13-18956-s005.zip › Figure 5E+F-ABA Raw data/0.5u-31.jpg]

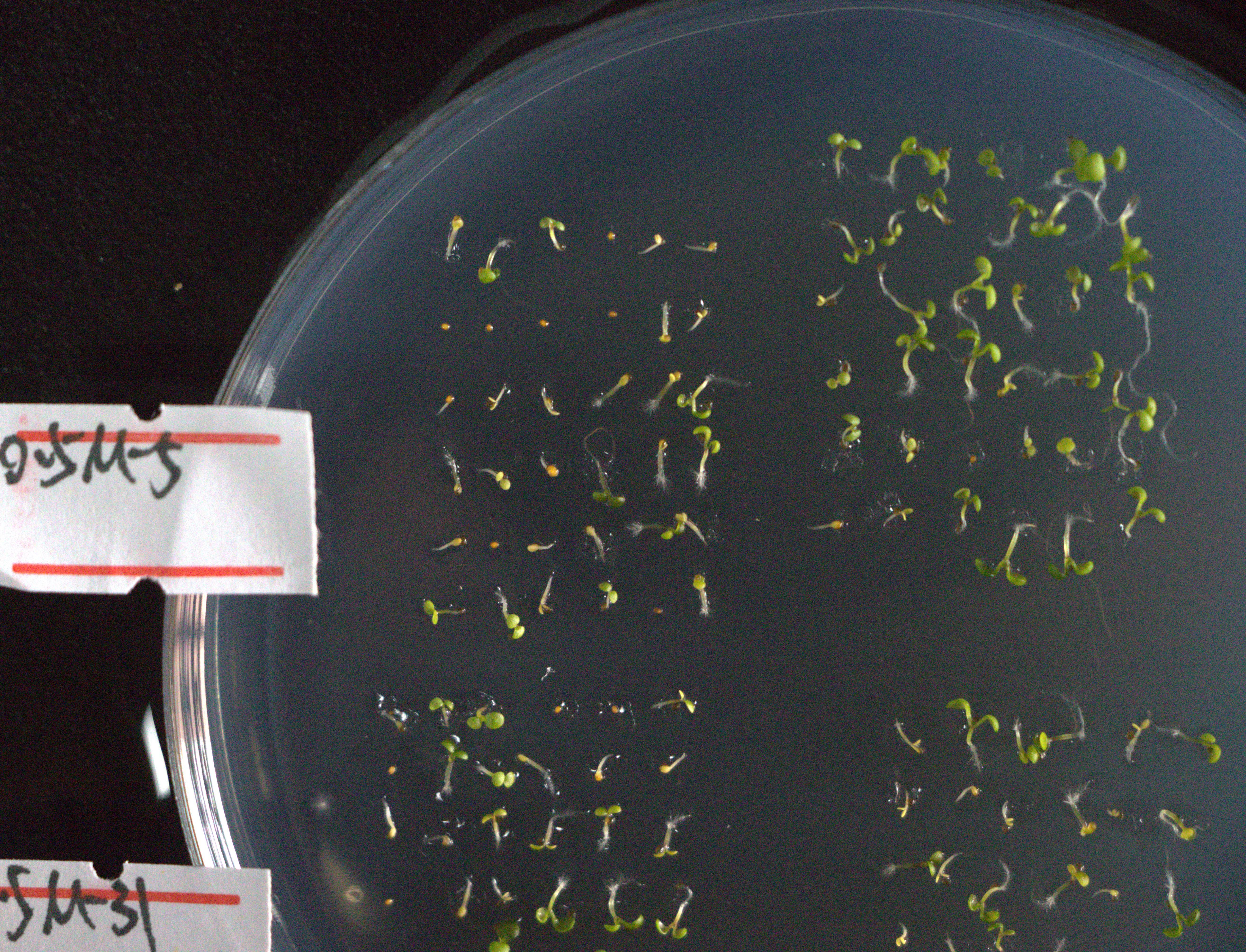

Supplement: Data S5 [file peerj-13-18956-s005.zip › Figure 5E+F-ABA Raw data/0.5u-5.jpg]

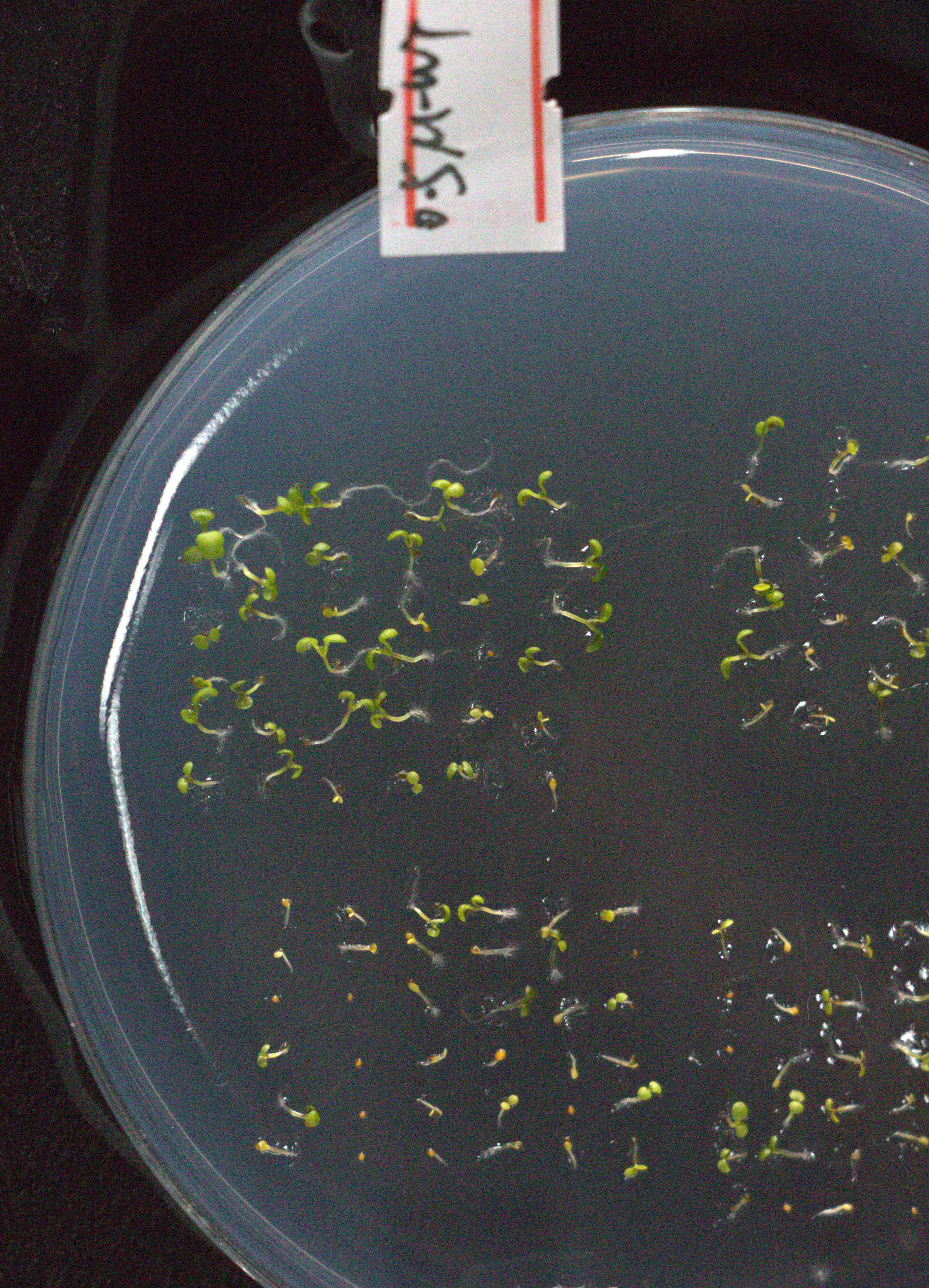

Supplement: Data S5 [file peerj-13-18956-s005.zip › Figure 5E+F-ABA Raw data/0.5u-wt.jpg]

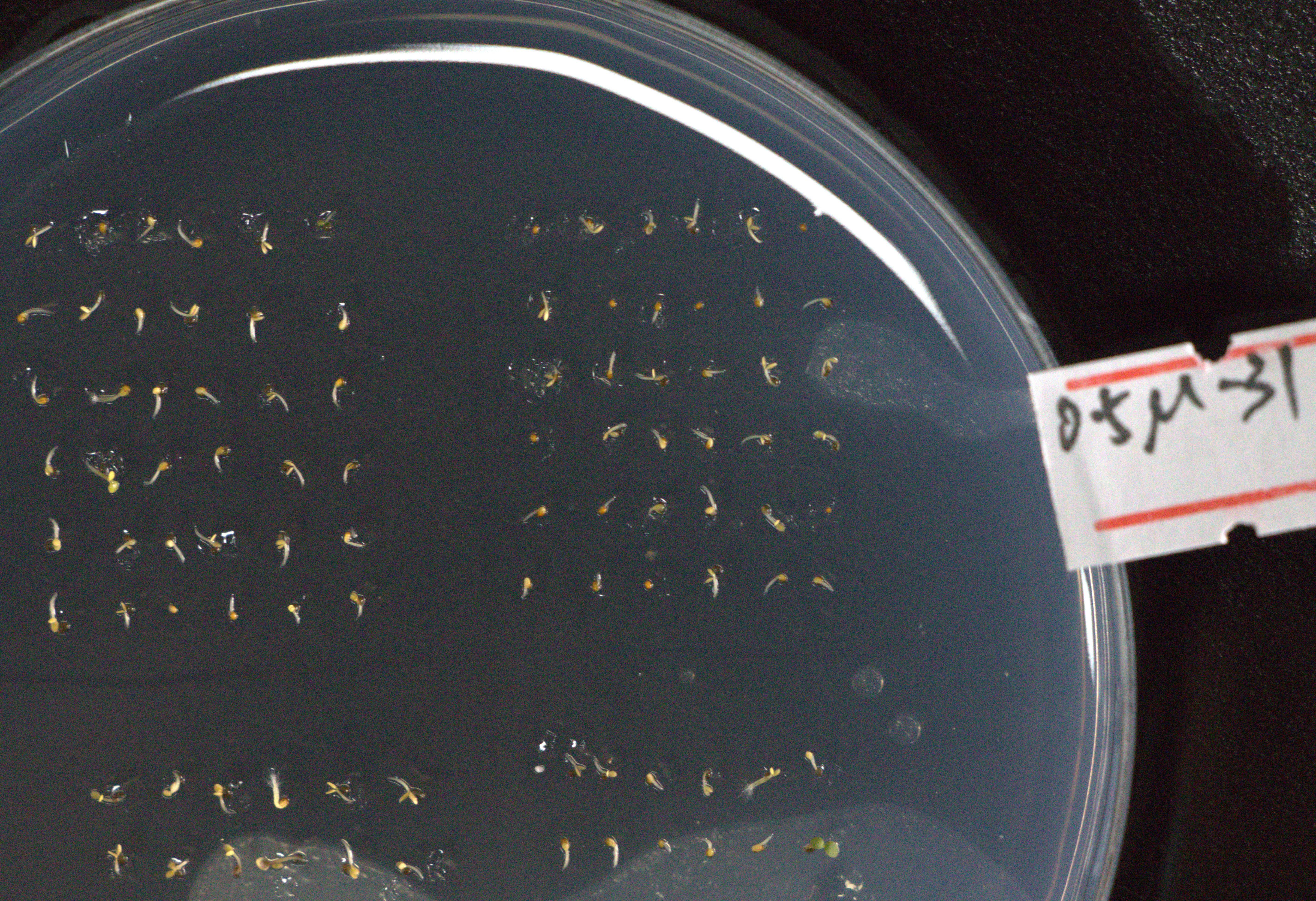

Supplement: Data S5 [file peerj-13-18956-s005.zip › Figure 5E+F-ABA Raw data/1u-31.jpg]

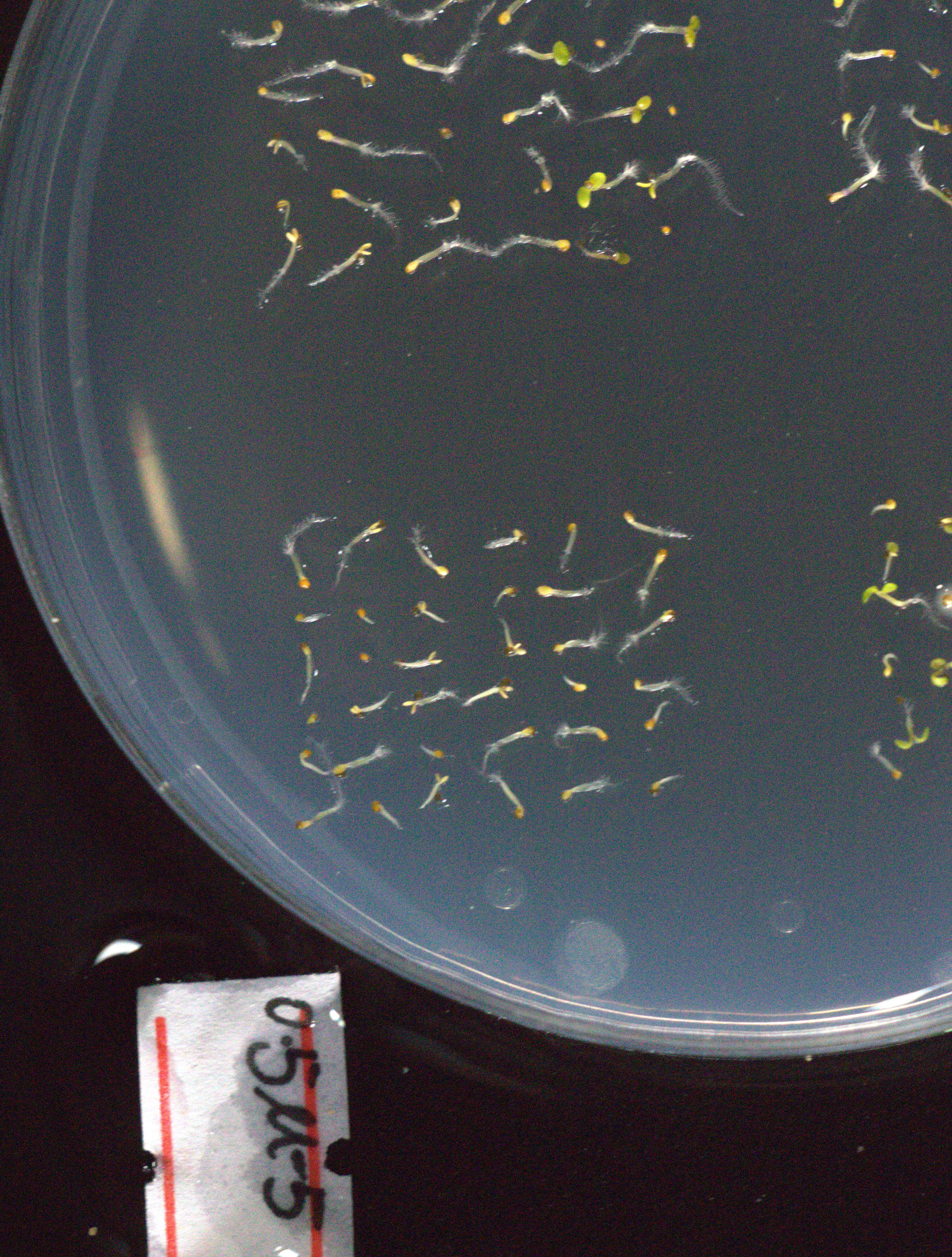

Supplement: Data S5 [file peerj-13-18956-s005.zip › Figure 5E+F-ABA Raw data/1u-5.jpg]

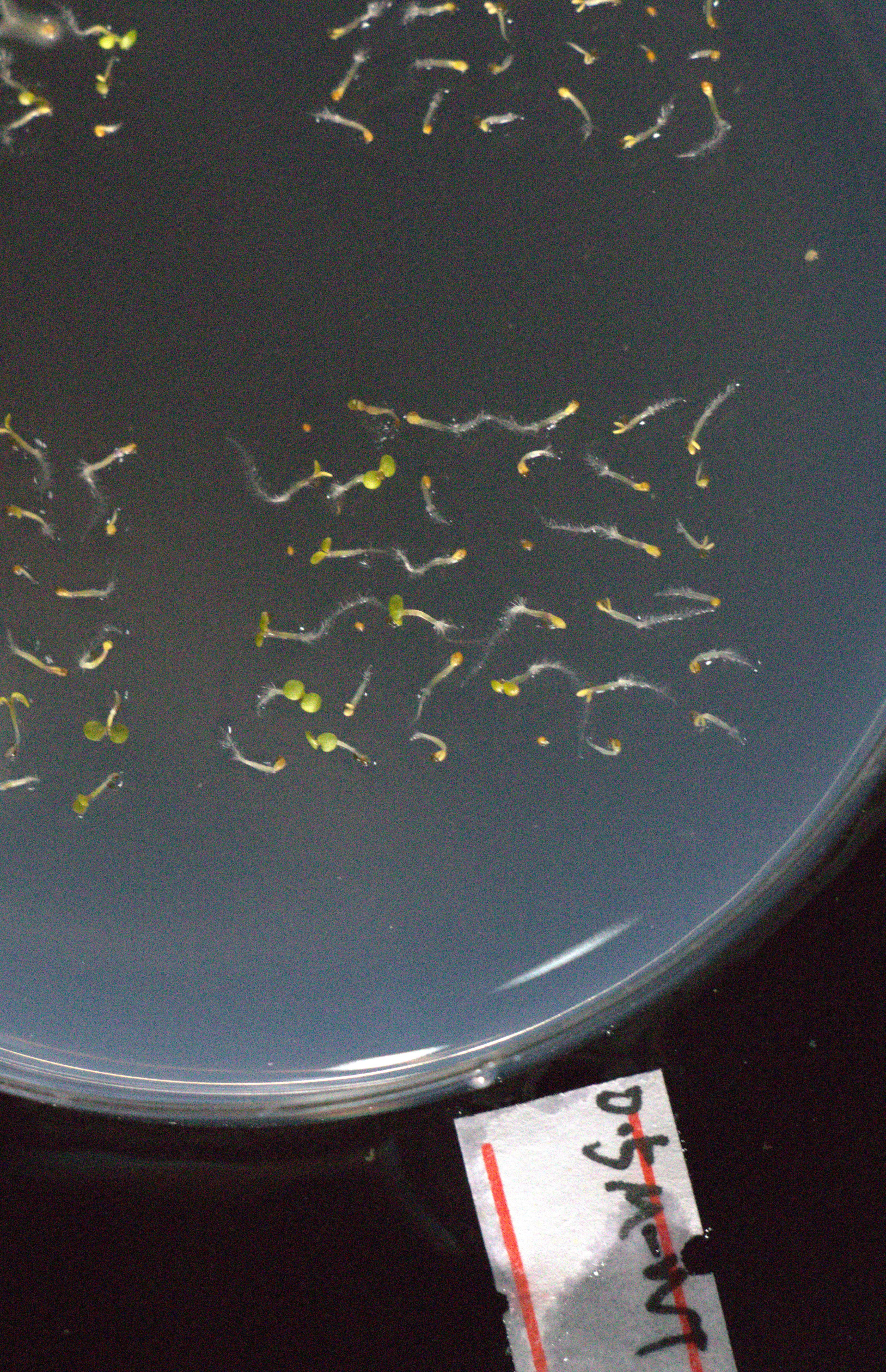

Supplement: Data S5 [file peerj-13-18956-s005.zip › Figure 5E+F-ABA Raw data/1u-wt.jpg]

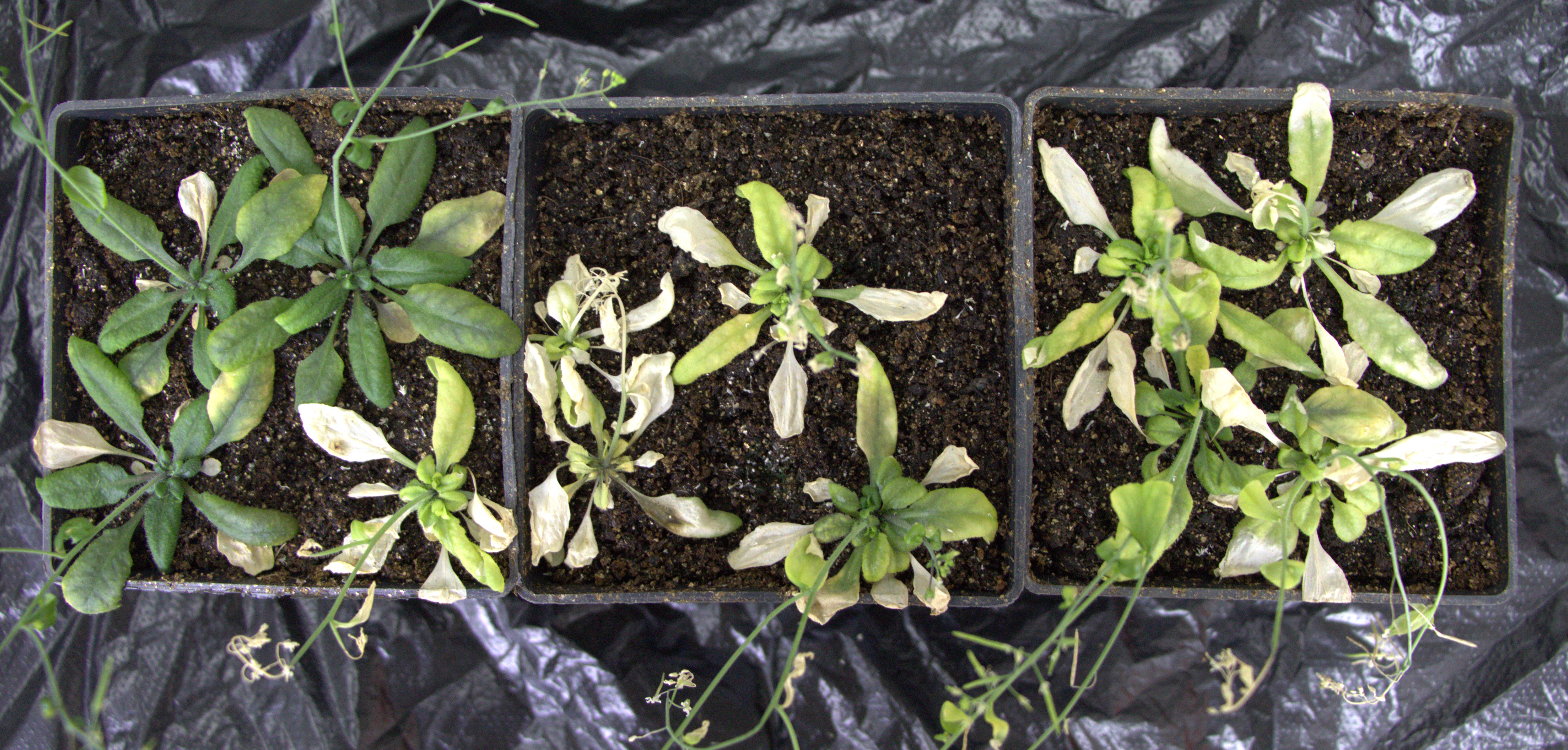

Supplement: Data S6 [file peerj-13-18956-s006.zip › Figure 6 Raw data/Figure 6A-300mM Nacl.jpg]

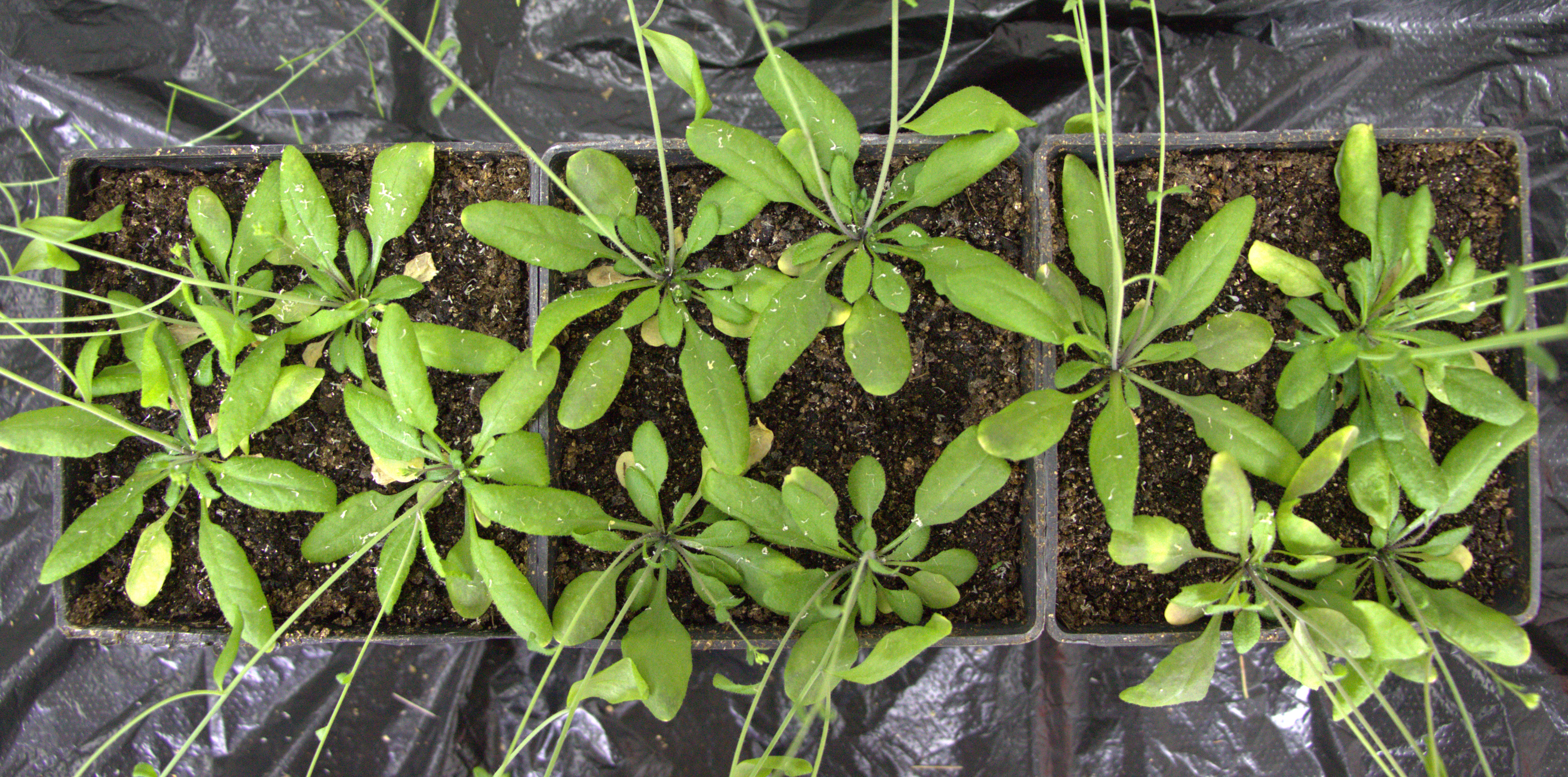

Supplement: Data S6 [file peerj-13-18956-s006.zip › Figure 6 Raw data/Figure 6A-Control.jpg]

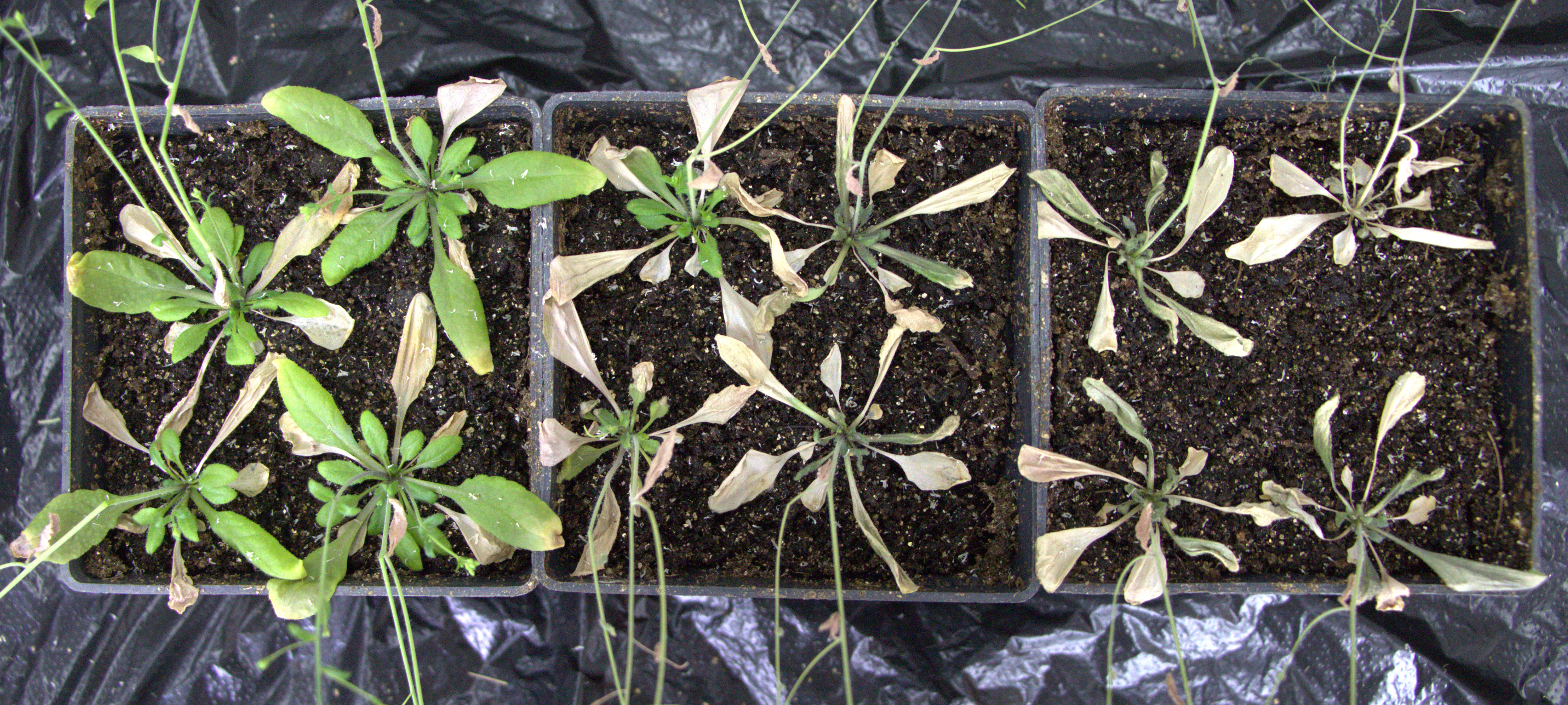

Supplement: Data S6 [file peerj-13-18956-s006.zip › Figure 6 Raw data/Figure 6C-Drought.jpg]
